# In memoriam- Dr. Robert Hodgson Heptinstall

**DOI:** 10.1186/s12882-021-02323-z

**Published:** 2021-05-03

**Authors:** Lorraine C. Racusen

**Affiliations:** Department of Pathology, The Johns Hopkins Medical Institutions, The Johns Hopkins University School of Medicine, 600 North Wolfe Street, 21287, School of Medicine, Baltimore, MD USA

**Keywords:** Pathology, Heptinstall, Memorium

## Abstract

This manuscript is a brief biography and reminiscence of Dr. Robert H. Heptinstall, a preeminent nephropathologist who had a major and formative impact on the field of nephropathology, and on nephrology in general. In his tour-de-force textbook of renal pathology, he brought the assessment of diseases of the kidney into the modern era. This textbook, now Heptinstall’s Textbook of Renal Pathology, will extend his seminal influence well into the future.

Dr. Robert Hodgson Heptinstall, one of the founding fathers of modern renal pathology, passed away on January 5, 2021 at the age of 100. “Heppy” was born in Keswick, Cumberland, England and received his medical education at Kings College London, Charing Cross Hospital Medical School, and London University. As a young physician, from 1944 to 1947 he served in the Royal Army Medical Corps in India, Burma, Siam and the Dutch East Indies as infantry medical officer and operational medical officer for special forces. During this time, he cared for prisoners of war, and was profoundly affected by this experience. In 1947 he returned to Charing Cross Hospital where he had initially trained as a surgeon. However, he decided to change his focus to pathology, a decision which led him to seek pathology training at St. Mary’s Hospital (Fig. 1).

**Fig. 1:**
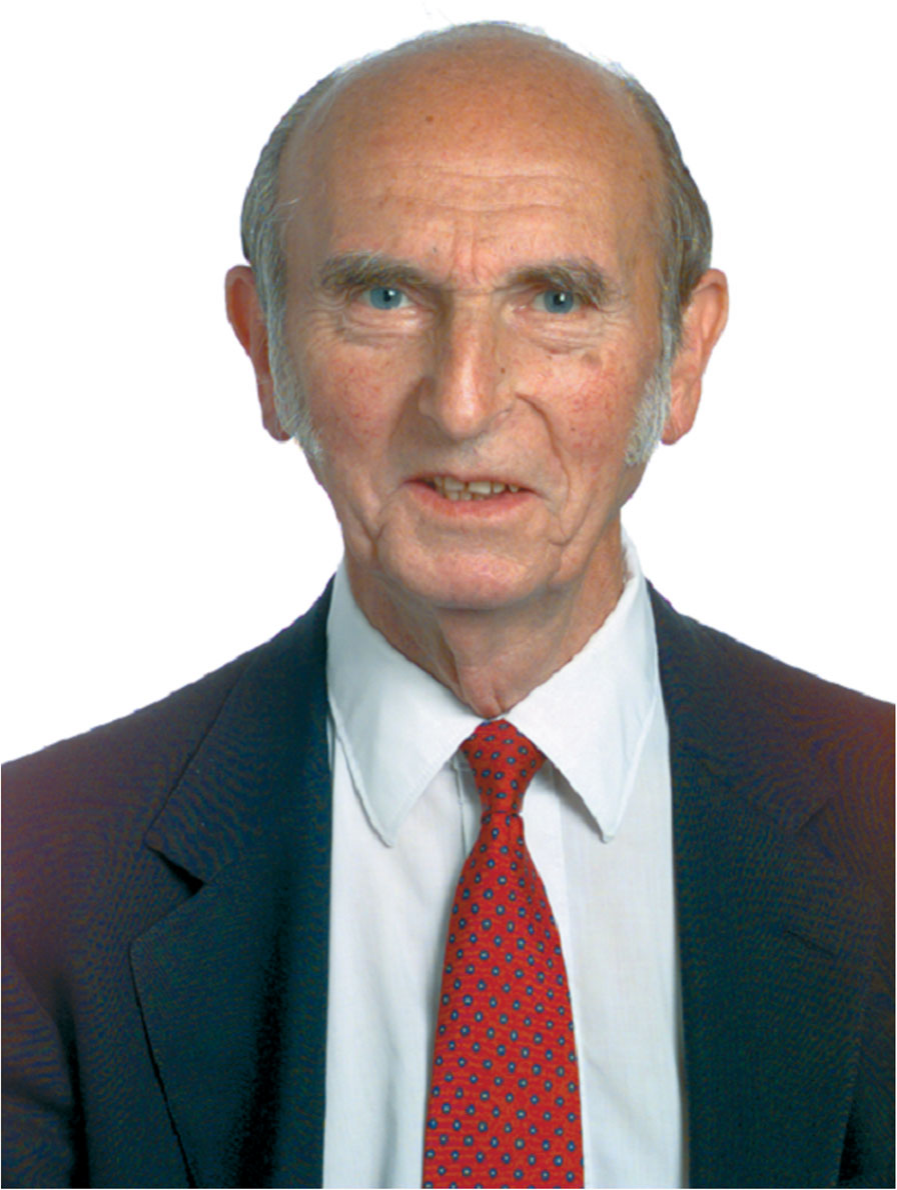
Dr. Robert Hodgson Heptinstall

In 1954, as an Eli Lilly Traveling Fellow, he did additional training in the Department of Pathology at the Johns Hopkins Hospital in Baltimore. He returned to England to St. Mary’s, serving as Honorary Consultant Pathologist until 1960; during this time he worked in the laboratory of Dr. Alexander Fleming. He then went to Washington University School of Medicine in St. Louis as Visiting Professor of Pathology. In 1962, he accepted an appointment as Associate Professor of Pathology at Johns Hopkins, rising to the rank of Professor in 1967. From 1966 until 1969, he served as acting Director of Pathology and acting Pathologist-in-Chief at Johns Hopkins. In 1969 he was appointed Baxley Professor of Pathology and Director of the Department of Pathology, a position which he held until 1988. In 1992, he became Distinguished Service Professor of Pathology.

Heppy trained at a pivotal time for nephrology and renal pathology, with much active basic research, and the introduction of the needle biopsy of the kidney to clinical practice. He became very interested in infections of the kidney and their sequelae, hypertension and atherosclerosis, and did seminal research in these areas. His research collaborators included Fred Germuth, Gary Hill, Kim Solez, Jean Olson, and Steve Wilson. He was a member of the Pathology A Study Section, and a member of the Pathology Training Grants Committee of the US Public Health Service. He served as Associate Editor of Nephron, and was on the Editorial Boards of the American Journal of Pathology, Kidney International, Medicine, Excerpta Medica, and Laboratory Investigation. From 1976 to 1981 he was co-editor of Laboratory Investigation with Dr. John Boitnott. He was a member of the United States and Canadian Academy of Pathology, the American Society of Nephrology (ASN), the International Society of Nephrology (ISN), and was a founding member of the Renal Pathology Club, now the Renal Pathology Society (RPS). Heppy was president of the RPS from 1980 to 1983. He was also an honorary member of the American Society of Clinical Pathology, and the Danish Society of Nephrology.

Heppy was the compleat academic pathology. He founded the renal biopsy service at Johns Hopkins, had an active research laboratory, and also loved to teach. Arguably his crowning contribution to education in renal pathology and nephrology was his textbook, The Pathology of the Kidney, first published in 1966. The book was a major advance in the field, as it incorporated the findings from clinical biopsies of the kidney as well as surgical and autopsy specimens. The writing of this text was a Herculean effort, occupying evenings and weekends for a long period of time. The book was extremely well-received, and a second edition followed, incorporating findings from immunofluorescence and electron microscopy as well as routine histology. This text, now called Heptinstalls Pathology of the Kidney, is in its seventh edition, with multiple editors and chapter authors, a vibrant part of Heppy’s legacy.

While Heppy was a dedicated renal pathologist, he was also very influential in the broader nephrology community. As noted, he served on editorial boards of a number of influential nephrology journals. In addition, he was active in professional nephrology societies. He held a number of offices in the American Society of Nephrology, and served as president of the ASN from 1972 to 1973. He was also active in the International Society of Nephrology, serving as vice-president of the society from 1981 to 1984. His high profile in the field, and his textbook, which is a major resource for nephrologists as well as pathologists, helped establish the close relationship between the two disciplines that continues to the day.

Another important part of Heppy’s life was his family. He married Ann Porter in the years after the war, and she was his life companion. Together they had seven children. Ann, as well as three of their children, predeceased him. The remaining children, Jonathan, James, Caroline and Gillian were very supportive in his later years. He also enjoyed his eight grandchildren and one great- grandchild. In retirement, Heppy lived independently until late in life, and remained active and engaged, avidly following medicine, sports and politics, and reading history. He retained a phenomenal memory as well as his wit and sense of humor. He was very convivial and enjoyed visiting with colleagues- he was a legendary story teller. His daughter Caroline Heptinstall Jones and her family provided a home for him in later years, and helped entertain friends and colleagues who came to visit him.

In addition to leadership roles in professional societies and journals, Heppy garnered many awards in his lifetime. These include the Gold Medal from the Danish Society of Nephrology, the David M Hume Memorial Award from the National Kidney Foundation, and the Jean Hamburger Award from the ISN. In his honor, in 2011 the Renal Pathology Society named their life-time achievement award in his honor. In 2020, the Johns Hopkins Department of Pathology established a fellowship in his honor. Heppy was truly “one of a kind”- we will not see his like again.

## Data Availability

Not applicable.

